# Functional Promiscuity of Homologues of the Bacterial ArsA ATPases

**DOI:** 10.1155/2010/187373

**Published:** 2010-10-20

**Authors:** Rostislav Castillo, Milton H. Saier

**Affiliations:** Division of Biological Sciences, University of California at San Diego, La Jolla, CA 92093-0116, USA

## Abstract

The ArsA ATPase of *E. coli* plays an essential role in arsenic detoxification. Published evidence implicates ArsA in the energization of As(III) efflux via the formation of an oxyanion-translocating complex with ArsB. In addition, eukaryotic ArsA homologues have several recognized functions unrelated to arsenic resistance. By aligning ArsA homologues, constructing phylogenetic trees, examining ArsA encoding operons, and estimating the probable coevolution of these homologues with putative transporters and auxiliary proteins unrelated to ArsB, we provide evidence for new functions for ArsA homologues. They may play roles in carbon starvation, gas vesicle biogenesis, and arsenic resistance. The results lead to the proposal that ArsA homologues energize four distinct and nonhomologous transporters, ArsB, ArsP, CstA, and Acr3.

## 1. Introduction

Arsenical species present threats to all organisms. The two predominant states of inorganic arsenic are arsenate [As(V)] and arsenite [As(III)]. As(V) disrupts the cellular energy machinery as a phosphate analog, uncoupling energy production. The even more toxic As(III) binds the sulfhydryl groups of cysteine residues and inactivates proteins [1]. A variety of defenses have evolved against arsenic toxicity. Plasmid- and chromosome-borne *ars* operons encode genes including *arsA*, -*B*,* -C*, -*D*, -*H*, -*I*, -*P*, and -*R*.

*arsC* encodes an arsenate reductase that transfers electrons to As(V), reducing it to As(III), the efflux pump substrate [2]. ArsD is a metallochaperone which sequesters arsenite and antimonite and transfers them to the ArsA ATPase increasing the apparent affinity of ArsA for its substrates and lowering the concentration of free As(III) and Sb(III) in the cytosol [3]. ArsH, which is related to NADPH-dependent FMN reductases, has been implicated in arsenic resistance, but its precise biochemical function is not yet known [4]. *arsP* encodes a putative transmembrane permease which may play an ill-defined role in arsenic resistance in some organisms [5].

ArsB is a 12 *α*-helix transmembrane spanning (TMS) pump extruding As(III) and Sb(III) [6]. Transport via ArsB can be energized by the pmf or by forming an oxyanion-translocating complex with the catalytic ArsA subunit, coupling ATP hydrolysis to efflux [7]. The characterized *E. coli* ArsA, a 583-amino acid (aa) ATPase, is composed of two homologous domains, A1 and A2, joined by a flexible linker. A1 and A2 are undoubtedly the products of a tandem intragenic duplication event. As(III) and Sb(III) form covalent bonds at the metal binding sites in A1 and A2, bringing the two domains together [8]. ArsA is allosterically activated by arsenite and antimonite binding, causing the nucleotide binding domains (NBDs) from each homologous half to come together to bind and hydrolyze ATP. Loss of either domain causes ArsA to lose catalytic function [9]. Prokaryotic ArsA has been extensively studied; however recent investigations of eukaryotic ArsA homologs, ASNA-1 (TRC40) and Arr4p, have furnished a variety of new roles for this family of proteins [10–15].

Previous studies have revealed that ATPases that energize macromolecular secretion can be found associated with a variety of evolutionarily distinct export systems. For example, the family of ATPases that energize bacterial conjugation (the transfer of DNA from a male donor to a female recipient) can also be found in a variety of other secretion systems. In addition to energizing conjugation via a Type IV secretion system, or its assembly, these ATPases can function in protein toxin secretion, bacterial pilus biogenesis, Type II protein secretion, and archaeal flagellar biogenesis. These facts reveal the promiscuity of ATPase energizers in the ultimate functional roles which have evolved over time [16]. Another example of functional promiscuity can be found in the ABC superfamily, where a single receptor can function with multiple transporters and a single transporter can utilize multiple receptors, thereby broadening substrate specificity (see the ABC superfamily; TC no. 3.A.1.1–34 in the Transporter Classification Database, http://www.tcdb.org/). Thus, “evolution is a tinker that cobbles together new functions from old ones, and the genome is a kind of parts bin of recyclable elements” (P. Z. Meyers).

Recently, established studies and our own analyses prompted further scrutiny of ArsA for uncharacterized roles in prokaryotes. We have conducted bioinformatic analyses using SEED, which revealed that different *arsA* homologues are often associated with genes for gas vesicles biogenesis and carbon starvation. Carbon starvation gene A (*cstA*) is often associated with an “auxiliary” genes *cstX*, and sometimes in archaea with *cstY*. *arsA* homologues are commonly present in operons that lack *arsB* but contain *acr3*, a gene encoding an evolutionarily distinct As(III) efflux pump [17]. We include phylogenetic analyses of ArsA, ArsB, Acr3, ArsP, CstA, ArsA, CstX, and CstY, demonstrating that some but not others apparently coevolved. We identify new *ars* operon determinants encoding CcdA, thioredoxin, and redox-active disulfide protein 2 homologues. Finally, we have investigated the extent to which *arsP* homologues are encoded in prokaryotic operons together with *arsA* homologues. Our results suggest that ArsA plays roles in addition to those previously recognized.

## 2. Materials and Methods

### 2.1. Phylogenetic Tree Construction

A TCDB [18–20] ArsA query sequence was used in a default cutoff PSI-BLAST [21] search of the NCBI protein database to identify homologues. These were then screened using MakeTable5 [22] with a 90% cutoff to remove redundancies, close sequences, and fragmentary sequences. The CLUSTAL X program [23] was used to create multiple alignments of the homologue sequences retained by MakeTable5, and TREEVIEW [24] was used to draw phylogenetic trees generated from CLUSTALX alignments. 16S and 18S rRNA sequences were obtained from NCBI.

### 2.2. Superfamily Analyses

The Superfamily Tree program SFT1 [22] provided the evolutionary relationships of ArsA to other homologues using the neighbor-joining method. Representative proteins for each family were selected following an NCBI BLASTP search for sequences with 30%–50% identity and *e*-values less than *e* ^−60^. These same sequences were analyzed using ProtPars, which uses the parsimony method to predict protein relationships [25].

### 2.3. TMS Predictions

To predict TMSs, the WHAT program [26] was used to plot hydropathy, and the AveHAS program [27] was used to derive average hydropathy, amphipathicity, and similarity plots. These updated programs were used with the default sliding window of 19 residues.

### 2.4. SEED Analyses

Genome context analyses were performed with the SEED comparative genomics database [28, 29]. This database can be found at http://theseed.uchicago.edu/FIG/SubsysEditor.cgi?page=ShowSpreadsheet&subsystem=CstA_Experiment and http://theseed.uchicago.edu/FIG/SubsysEditor.cgi?page=ShowSpreadsheet&subsystem=Arsenic_and_Antimonite_Resistance. Proteins were excluded from these analyses if they were not represented in the SEED database, or if they occurred in *ars* operons with low frequency. These included redox proteins of unknown function and potential modifying enzymes such as acetyl-transferases. Operons were delineated based on SEED predictions. The putative functions of proteins were predicted by operon context analyses which included functional assignments based on coregulated genes. To study coevolution between proteins, we used SEED to select pairs of proteins that were encoded within the same operon.

### 2.5. MEME Analyses

The MEME program [30], with default settings, was used to search for ungapped conserved residue motifs with the ArsA homologues that we identified using PSI-BLAST as our training set.

## 3. Results

### 3.1. The Phylogeny of ArsA and arsA Gene Associations

**Figure 1:**
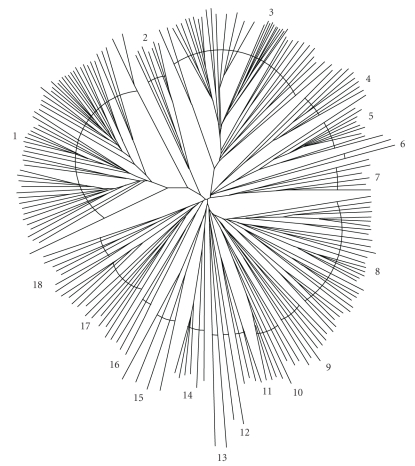
Phylogenetic tree of full length prokaryotic and eukaryotic ArsA homologues. The CLUSTAL X program [23] was used to create a multiple alignment of the protein sequences, while TreeView [24] generated the tree. The phylogenetic clusters are labeled 1–18 clockwise. Specific proteins can be found in Table 1, first according to cluster number and then according to position within the cluster. The dendrogram is shown in Figure S1.

Using *E. coli* ArsA as the query sequence, a PSI-BLAST search of the NCBI protein database brought up homologues which were screened using MakeTable5 [22] with a 90% cutoff to remove redundancies, close sequences, and fragmentary sequences. These homologues are listed in Table 1. The CLUSTAL X program [23] was used to create a multiple alignment, and TREEVIEW [24] was used to draw the phylogenetic tree (Figure 1). The tree is divided into 18 clusters. These proteins can be found in Table 1, first, according to the cluster number, and second, according to their positions within the cluster.

To identify the potential functions of the proteins of each cluster, we used SEED to perform genome context analyses for organisms in the SEED genome database. Based on the pattern of association of the genes in each of the clusters, we have identified connections between various *arsA* homologues and other previously unrelated genes.

*Cluster 1* is composed of 55 bacterial and archaeal ArsA homologues, including the well-characterized *E. coli *ArsA protein, with an average size of 589 aas. Of the 24 cluster 1 proteins in SEED, 19 are encoded within operons containing homologues of *arsB*, *acr3*, and/or *arsP*. Of the remaining five, four are next to an *arsD* homologue, three are located in operons with *arsR *homologues, and three have *arsB*, *acr3*, or *arsP* homologues elsewhere in the genome. The high degree of association of *arsA* homologues in cluster 1 with other *ars* operon determinants and the similarities in sizes between these proteins and the *E. coli* ArsA suggest that most Cluster 1 proteins function as energizers equivalent to the *E. coli *ArsA protein.

*Cluster 2* is composed of seven archaeal ArsA homologues. SEED analysis was performed on the six members of this cluster included in the SEED database. Of these, none is found in the context of any of the typical *ars* operon genes, but four have *acr3* or *arsP* homologues elsewhere in the genome. The tightly clustering proteins, Mja Mma2 and Mae2, are the three which have *arsP* genes elsewhere in the genome, while Msm1, clustering separately from the other three proteins, has an *acr3* and an *arsP* elsewhere in the genome. The remaining proteins are encoded in genomes that lack any other *ars* transporter genes. Cluster 2 ArsA proteins are composed of a single ArsA domain with an average size of 337 aas. Thus, if they function catalytically like the *E. coli *ArsA protein, they must dimerize. With no consistent pattern of associated genes, the functions of cluster 2 ArsA homologues remain unknown.

Thirty-nine eukaryotic ArsA homologues comprising *Cluster 3* have an average size of 345 aas. SEED analysis is only applicable for the *S. cerevisiae* genome; however, inspection of the region surrounding the *arsA* homologue did not identify functionally relevant proteins. Published evidence identifies these proteins as Arr4p and ASNA-1 homologues. While Arr4p from *Saccharomyces cerevisiae* is a member of the GET complex which is involved in trafficking from the Golgi apparatus to the ER and posttranslational insertion into the ER [14], homologues have been implicated in As(III) and Sb(III) resistance [15], metal and heat tolerance [13], regulation of Gef1p to prevent copper accumulation [12], and regulation of insulin secretion [10]. Based on these results, we presume that these single domain ArsA homologues serve a diversity of biological functions, either energizing or regulating various transport processes.

*Cluster 4* consists of eight bacterial ArsA homologues. Four of these are present in SEED. Each of these four genes is in an operon together with *gvpFGJKLMNOW*, encoding proteins involved in gas vesicle biogenesis, as well as a heat shock protein Hsp20 [31]. None of these four genes is present in an operon with other *ars* gene homologues, although *acr3* and *arsP* homologues are located elsewhere in three of these genomes. The average size of Cluster 4 proteins is 637 aas, similar to that of the *E. coli* ArsA. These observations suggest that these two domain homologues may function to energize some aspect of gas vesicle biogenesis.

*Cluster 5* contains ten eukaryotic ArsA homologues with an average size of 401 aas. The functions of these proteins may be similar to the functions of those in Cluster 3, but no literature exists elucidating the roles of these ArsA homologues.

The two bacterial ArsA homologues of *Cluster 6* have an average size of 645 aas. A SEED analysis revealed that the gene encoding Aba1 but not Mxa1 is associated with gas vesicle genes.

*Cluster 7* contains six archaeal ArsA homologues with an average size of 342 aas. Three proteins from this cluster are in the SEED database. Nph1 is encoded by a gene present in an operon with the carbon starvation induced genes, *cstA* and *cstX*. The other two homologues are not associated with genes that provide clues as to function.

*Clusters 8–13* all consist of single domain proteins with average sizes of 396, 389, 410, 424, 361, and 292, respectively. Representatives of four of these six clusters were present in SEED, and in every such case, the genomic content was analyzed. In no case did these analyses provide clues as to function. In a few cases, *acr3 *and *arsP *genes were found elsewhere in the genomes, but not in close proximity to the genes encoding ArsA homologues. Thus, in these clusters, we could not provide functional predictions.

*Cluster 14* contains six archaeal ArsA homologues with an average size of 327 aas. Three of these are in SEED. All three are in operons encoding CstA homologues, a single TMS (~90 aas) CstX and a single TMS (~210 aas) CstY. None is found in operons with *ars* operon determinants. Based on observations from Clusters 7 and 14–18, ArsA homologues that are associated with a CstA and a CstX tend to have single ATPase domains and a size of ~330 aas. The evidence suggests that proteins from this cluster function with CstA, CstX, and CstY. While CstA is a putative transporter, CstX and CstY may be auxiliary subunits [32–34]. Possibly the ArsA homologue energizes transport via CstA, thereby influencing the carbon starvation response.

The four bacterial and archaeal ArsA homologues of *Cluster 15* have an average size of 336 aas. All four are encoded in operons with CstA and CstX. These two pairs of ArsA homologues are adjacent genes in two organisms with a sequence identity of 31% for Par and 25% sequence identity for Sth. The context and size of the proteins in this cluster indicate that they function with CstA and CstX (but not CstY) homologues.

Of the three bacterial ArsA homologues in *Cluster 16*, two are in SEED. One is encoded within an operon containing CstA and CstX, but the other is not found in the context of functionally significant genes. The average size of the three ArsA homologues, 306 aas, and the context of one ArsA encoding gene suggest that this cluster is composed of proteins functioning with CstA and CstX in the carbon starvation response.

There are 12 bacterial and archaeal *arsA* homologues in *Cluster 17* with an average protein size of 326 aas. Nine are found in SEED. All are located in gene clusters also encoding CstA and CstX. Interestingly, one ArsA-like protein is 426 aas long. The extra residues, at the N-terminus, showed no sequence similarity with anything in the NCBI database. The context and average size of the genes encoding these proteins indicate that this cluster, like Cluster 16, functions with CstA and CstX.

*Cluster 18* is composed of seven bacterial ArsA homologues with an average size of 336 aas. Of the four found in SEED, all are encoded by genes in operons that also encode CstA and CstX.

Thus, Clusters 14–18 and possibly 7 (based on one example) may all be concerned with carbon starvation responses, functioning in conjunction with CstA, CstX, and CstY in cluster 14.

### 3.2. CstA Protein Associations

Using SEED, we have identified 26 instances in bacteria and archaea where CstA is associated with an ArsA homologue. In 24 of these instances CstX is found with CstA, but it is missing in one bacterium and one archaeon. CstX is never present without CstA. Five archaea analyzed also have CstY encoded within the cluster containg ArsA, CstA, and the CstX homologues. We suggest that CstA functions together with the ArsA and CstX homologues. Possibly CstX couples CstA-catalyzed transport to ArsA-dependent ATP hydrolysis as demonstrated for other transport systems [29]. It is important to note that these operons do not contain an *arsR* and thus are not likely to be regulated by the usual mechanism in response to the arsenite concentration.

**Figure 2:**
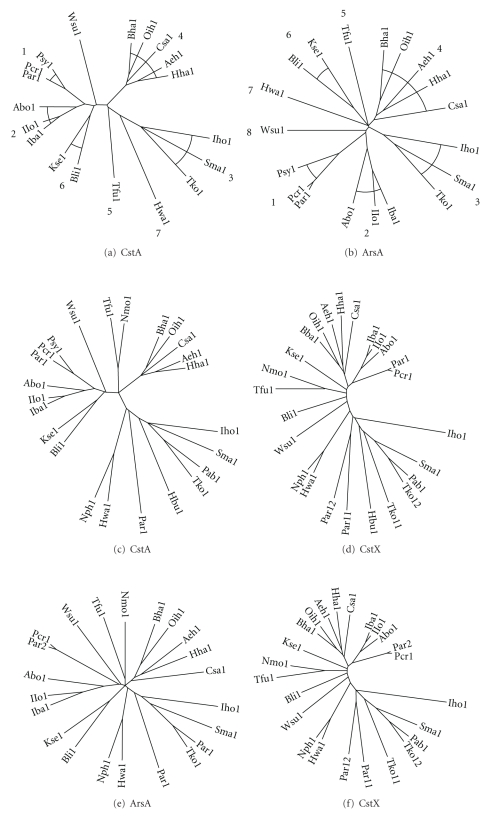
Phylogenetic trees showing the coevolution of (a) CstA and (b) ArsA homologues, (c) CstA and (d) CstX homologues, and (e) ArsA and (f) CstX homologues, each pair being encoded within the same operons. The methodology was as described in Figure 1. Cluster numbers are assigned counterclockwise in (b) and retained in (a). Paralogues are distinguished with a second digit, either 1 or 2, in the protein abbreviations.

Phylogenetic trees were generated for those CstA, CstX, CstY, and ArsA homologues that occur together within the same operons. Trees for the CstA and ArsA homologues are shown in Figures 2(a) and 2(b), respectively, for CstA and CstX homologues in Figures 2(c) and 2(d), respectively, and for ArsA and CstX homologues in Figures 2(e) and 2(f), respectively. Comparing Figures 2(a) and 2(b), we see that clustering patterns correspond within experimental error. Thus, Clusters 1 and 2 branch more closely from each other than they do to any other branch. These and all remaining clusters have the same protein compositions on the two trees. Most of these clusters branch from points near the centers of these trees, as is particularly apparent in Figure 2(b). It is interesting to note that the CstA tree shows tighter clustering than observed for the ArsA tree. Moreover, within the three-protein cluster 3 and the five-protein cluster 4, branching patterns are the same in both trees. We therefore conclude that these two families of proteins have coevolved.

Comparing the phylogenetic tree for CstX (Figure 2(d)) with that for CstA (Figure 2(c)), we see that almost all proteins show excellent correspondence in the two trees. For example, at the bottom of both trees, we find the same clustering patterns for the two sets of proteins, except that each organism possesses a single CstA, but in two cases, there are two paralogous CstX proteins, Par11 and Par12, and Tko11 and Tko12. In both cases, it appears that the novel paralogue arose relatively early. Interestingly, Tko12 exhibits striking similarities with Pab1 and Sma1, while Tko11 has no such close ortholog (see Figure 2(d)). The top halves of the two trees similarly show clustering patterns largely consistent with coevolution. However, there are a couple of potential inconsistencies. Bli1 and Kse1 cluster together in Figure 2(c), but not in Figure 2(d). This observation is anomalous because in all other subclusters shown in these two trees, the proteins show shorter branch lengths in Figure 2(d) compared to those in Figure 2(c). Additionally, Wsu1 stems from the center of Figure 2(d) tree but clusters more tightly with the top half of the tree in Figure 2(c). We therefore conclude that CstA and CstX evolved in parallel with just two potential exceptions. The results are consistent with coevolution and therefore with cofunctioning, possibly involving direct interaction.

Comparing the CstX phylogenetic trees (Figure 2(f)) with that for ArsA (Figure 2(e)), we see that almost all proteins in one tree correspond in position to those in the other tree. It appears that ArsA has diverged in sequence more rapidly than has CstX, accounting for the greater branch lengths in the ArsA tree (Figure 2(e)). Potential exceptions are observed when Figures 2(e) and 2(f) are compared or when Figures 2(c) and 2(d) are compared. Thus, we observe the same anomaly regarding the inversion of Wsu1 with Bli1 as well as the clustering of Kse1 with Bli1 in Figure 2(e) but not Figure 2(f). The correspondence between Figures 2(a) and 2(b) proved to be greater than that between Figures 2(c) and 2(d) or Figures 2(e) and 2(f). We suggest that CstX homologues might interact with both the ArsA and CstA homologues, possibly because CstX serves as the linker between the other two proteins.

An additional protein found within the operons of five archaea is the CstY protein. The CstY tree showed branching patterns almost identical to those of the CstA tree. Four of these five operons include the *arsA* gene, and except for the absence of ArsA in *Hyperthermus butylicus* DSM 5456, we see that CstY also appeared to coevolve with CstA and ArsA. In like fashion, CstY seems to have coevolved with CstX except for the duplication in *Thermococcus kodakarensis* KOD1 (data not shown).

### 3.3. Gas Vesicle Biogenesis Proteins

Several ArsA homologues were encoded within clusters of genes concerned with gas vesicle biogenesis. These gene clusters were identified in four different phyla. Two organisms from the Bacteroides/Chlorobi phyla, *Polaribacter irgensii *23-P and *Pelodictyon luteolum* DSM 273 possess gene clusters where the *arsA* homologue colocalized with the following genes: *gvpNLFGJKM* and *hsp20*. Although the genes represented in the two clusters from the above organisms were the same, five and four copies of the paralogous *L* and *F* genes [35], and two and four copies of the *M* gene were present in these two clusters, respectively. Most similar to these clusters was the cluster from the *δ*-Proteobacterium, *Geobacter uraniireducens* Rf4. This cluster differs from the other two in that there are paralogues of the *L*/*F* genes, two paralogues of the *M* gene and an additional gene, the *O* gene. The cyanobacterial cluster, represented by *Anabaena variablis* ATCC 29413 and *Nostoc* sp. PCC 7120, also resembles that found in the previously described organisms, except that *L*/*F* genes are present in single copies, the *M* and *hsp20* genes are lacking, and an additional gene, the *W* gene, is present. Finally, in the acidobacterial phylum the *arsA* homologue is accompanied by two copies of the *L*/*F* genes and one copy each of the *J*, *K*, and *M* genes.

Very little is known about the genes involved in gas vesicle biogenesis. However, all of the genes described above are soluble proteins except for GvpG and GvpM which each has a single TMS. GvpG, of about 126 aas, has an N-terminal TMS, while GvpM, of about 100 aas, has its TMS centrally located. GvpN is an ATPase of the AAA+ superfamily with both the Walker A and Walker B motifs. This protein has been reported to increase the production of gas vesicles [36]. GvpL/F have been reported to be nucleation proteins, functioning in oligomerization of the protein complex [31]. The GlpW protein (228 aas) is distantly related to the GvpL/F proteins. Finally, a BLAST search revealed that among the homologues of GvpK, one, of ~158 aas, possesses a C-terminal domain linked to an aspartyl nucleophile Haloacid Dehalogenase domain. Similar to the carbon starvation operons, the gas vesicle operons do not contain *arsR.*

### 3.4. ArsB Protein Associations

**Figure 3:**
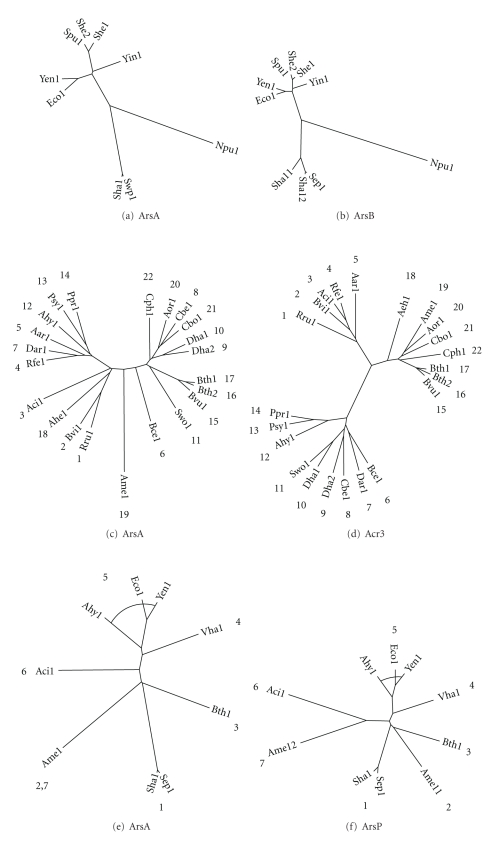
Phylogenetic trees showing the coevolution of (a) ArsA and (b) ArsB homologues, (c) ArsA and (d) Acr3 homologues, and (e) ArsA and (f) ArsP homologues, each pair encoded within the same operons. Numbers are assigned in (d) and retained in (c). Numbers are assigned counterclockwise in (f) and retained in (e). Paralogues are distinguished with a second digit, either 1 or 2, in the protein abbreviations. gi numbers are provided in Table 1.

The phylogenetic trees for ArsA and ArsB (Figures 3(a) and 3(b), resp.) include proteins encoded in operons that include both homologues. These two trees reveal nearly identical clustering patterns. Thus, in both trees, there are three major branches, and within each cluster the corresponding proteins can be found. The Npu1 homologues branch distantly from the others, while the Sha and Sep proteins cluster tightly together. There are two Sha paralogues in the ArsB tree, suggesting that a recent gene duplication event only for these two proteins occurred for ArsB but not for ArsA. Examination of the operon encoding these paralogues revealed that the duplication encompassed not only the *arsB* gene but also the adjacent *arsR* and *arsC* genes which flank *arsB*. Similar intergenic spacing is observed for the two duplicated DNA segments. Finally, in the last cluster, we again see correspondence within the two trees. While Yin is distant from the other proteins, the Spu and She orthologues cluster tightly together as do the Yen and Eco proteins. The ArsB proteins in the third cluster are much more tightly clustered than are the ArsA proteins, suggesting that the latter have diverged in sequence more than the former.

ArsA/ArsB pairs not encoded within the same operon were added to the proteins represented in Figures 3(c) and 3(d). Examination of Figures S2(a) and S2(b) shows that the Sha1-3E and Sep1-2E ArsB paralogues may have coevolved with the ArsA homologues. However, these two ArsB homologues are clearly more divergent in sequence than the two other paralogues in this organism, Sha1-1 and Sha1-2. Therefore, we suggest that these two ArsB homologues have diverged in sequence more rapidly than their paralogues. We similarly examined the ArsB homologues Yen1-2E and Spu1-2E. In contrast to the results obtained with Sha1-3E and Sep1-2E, these paralogues appear to have arisen by a recent gene duplication event, and none of these paralogues have diverged in sequence more rapidly than the others. It is possible that these ArsB homologues are fully functional and that both sets of paralogues can be energized by the ArsA proteins. None of the other proteins in Figures S2(a) and S2(b) appear to have coevolved; (see Figures S2(a) and S2(b) in Supplementary Material available online at doi:10.1155/2010/187373).

### 3.5. Acr3 Protein Associations

By analyzing the Arsenic and Antimonite Resistance SEED subsystem spreadsheet, we identified 33 instances in 28 bacteria and archaea in which an *ars* operon contains an *arsA* homologue in a genome lacking an *arsB* homologue. We also found 23 instances in 20 bacteria in which *arsA* genes are located in an operon encoding Acr3 but not ArsB, and 9 instances in 9 bacteria where *acr3* is in the same operon as *arsA*, but *arsB *and *arsP* are nowhere in the genome. These data indicate that some ArsA homologues from cluster 1 may function with Acr3, the only other characterized pump in the operon, as an ATPase energized As(III) efflux pump. These analyses suggest that these ArsA homologues may have functions independent of their role in the ArsAB translocation complex.

Trees for the ArsA and Acr3 proteins included in the same operons were derived as discussed above (Figures 3(c) and 3(d), resp.). However, in contrast to the two examples described above, there appeared to be very little correspondence between these two trees. The only common clustering patterns were observed between Ahy1, Psy1, and Ppr1 which cluster similarly as do Bth1, Bth2, and Bvu1. However, this is where common clustering patterns end. In Figure 3(c), we find the former cluster loosely associated with proteins 7 through 11, while in Figure 3(d), these proteins cluster with proteins 4, 5, and 7. Moreover, proteins 8, 9, and 10 are together with proteins 6 and 7 in Figure 3(c), but they cluster with 20, 21, and 22 in Figure 3(d). Further examination of these two trees reveals that there are no additional similarities, clearly demonstrating that these two sets of proteins did not coevolve. The implication of this finding is that these proteins either do not function together, or that their associations do not depend on strict protein-protein interactions. It should be noted that a lack of coevolution of proteins that interact physically to form a functional complex is rare but has been observed [32–34].

When examining *acr3* and *arsA* pairs (Figures S2(c) and S2(d)) that map separately within the same genomes, we found very few phylogenetic correlations indicative of coevolution. However, several examples of late gene duplication were detected where the two paralogues showed high degrees of sequence identity. Within the ArsAs there are two examples, while in Acr3s three such paralogous pairs were identified.

### 3.6. ArsP Protein Associations

A previous study has noted an instance of an *arsP* gene, of unknown function, in an *ars* operon [5]. The Arsenic and Antimonite Resistance subsystem spreadsheet identified 61 instances, including the previously characterized *arsP*, in 52 bacteria and archaea where *arsP* is found in an *ars* operon. Our data demonstrate that genes coding for ArsP homologues are widely distributed in bacteria and archaea. In addition, there are two instances, one archaeal and one bacterial, in which *arsP* is in the same operon as *arsA* in the absence of either *acr3* or *arsB*, tentatively suggesting a functional link between ArsA and ArsP. There are two distinct types of *arsP*, one encoding a ~300 aa 8 TMS putative transporter, another encoding a ~400 aa 8 TMS putative transporter with a ~100 aa hydrophilic insertion between the fourth and fifth TMSs. There are no clues as to the function(s) of ArsP homologues.

As described above, we constructed phylogenetic trees for the ArsA (Figure 3(e)) and ArsP (Figure 3(f)) homologues which coexist in operons. Comparison of these two trees reveals that clustering patterns are the same within experimental error with one exception. This exception refers to the presence of two distant Ame paralogues in the ArsP tree but not in the ArsA tree. Excluding this paralogous arrangement, due to an extragenic duplication event, we find that the Sha and Sep proteins cluster tightly together (Cluster 1), that the Ahy, Eco, and Yen proteins cluster somewhat less tightly together (Cluster 5), and that branch 4 clusters loosely with Cluster 5 in both trees. Similarly, Clusters 1, 2, and 3 are more closely related to each other than they are to the other clusters on both trees. Finally, branch 6 is positioned between these two major clusters on both trees. The results show that *arsB* homologues that occur within operons that also contain *arsP* homologues coevolved. Overall, ArsP, ArsB, CstA, and CstX coevolved with different ArsA homologues, although Acr3 did not (see Section 4). It is interesting to note that within Cluster 1, the ArsP homologues are more distantly related to each other than are homologues in the ArsA tree, but that the opposite is true for all other corresponding pairs of proteins examined.

ArsA/ArsP pairs not encoded within the same operon were identified in the genomes of several organisms not represented in Figures 3(e) and 3(f). Examination of Figures S2(e) and S2(f) shows that Vfi1 ArsA and Vfi1E ArsP appear to have coevolved, as is also true for the clusters including Dha1E, Dha2E, and Aor1E. However, Spu1E ArsP, within the same cluster as Vha1 and Vfi1E, does not appear to have coevolved with Spu1 ArsA. In fact, none of the other pairs of proteins in Figures S2(e) and S2(f) appear to have coevolved. It therefore seems unlikely that these proteins function together.

### 3.7. ArsD Protein Associations

The ArsD protein is a metallochaperone protein that is encoded in many *ars *operons that also encode ArsA. In fact, these two genes are frequently found adjacent to each other in these operons. We therefore conducted coevolutionary analyses of these two proteins as illustrated in Figures S3(a) and S3(b). Most of the 25 pairs of proteins showed similar clustering patterns, with a few exceptions. For example, Bce1 clusters with Cbo1, Cbe1, and Aor1 in the ArsD tree, but not in the ArsA tree. Furthermore, Gur1 clusters with Aeh1 and Bvi1 in the ArsA tree but not in the ArsD tree. Branch lengths are comparable in the two trees, although some of the clusters such as the two large clusters at the top and bottom of the trees show that the branch lengths are shorter for the ArsA than the ArsD homologues, indicating that ArsA is less sequence divergent than ArsD. This, however, was not uniformly observed. Another difference was observed in the top cluster where in the ArsD tree, Psp1 and Ahy1 cluster loosely together and Vfi1 and Ppr1 cluster more tightly together. In contrast, in the ArsA tree, Psp1 clusters fairly tightly with Ppr1 and more loosely with Vfi1, while Ahy1 occurs between the Asp1/Rfe1 cluster and Vfi1. These differences appear too great to be accounted for by experimental error. In conclusion, it appears that ArsD and ArsA have coevolved in the majority of cases, but a significant fraction of these protein pairs showed divergence suggestive of horizontal gene transfer or nonuniform rates of sequence divergence. We suggest that these proteins did not always coevolve.

### 3.8. ArsH Protein Associations

Only four of the *arsA* containing operons in SEED proved to have *arsH* genes. When the only four pairs of these proteins were examined from a phylogenetic standpoint, they showed no correspondence. For example, Yen1 and Kpn1 ArsH proteins cluster together but the corresponding ArsA proteins are on opposite sides of the ArsA tree. Although very few homologues were available for analysis, the results imply that ArsA and ArsH did not coevolve.

### 3.9. ars Operons Encoding CcdA, Thioredoxin, and Redox-Active Disulfide Protein 2

We have identified three new *ars* operon determinants, CcdA, thioredoxin, and redox-active disulfide protein 2, coding for components of a putative arsenate reductase system. Using SEED, we found 8 instances in 7 organisms where these three redox genes are present in *ars* operons. In 7 of these 8 instances, *arsC* genes are located in the operons with these determinants. CcdA is a DsbD homologue, which might catalyze electron transfer from the cytoplasm to the periplasm [37]. However, CcdA lacks the thioredoxin-like domain of the *E. coli* DsbD, suggesting that thioredoxin-like proteins present in the *ars* operons may donate electrons to CcdA. The redox-active disulfide protein 2 has a thioredoxin-like conserved domain and is a disulfide oxidoreductase. The thioredoxin-like protein and redox-active disulfide protein 2 undoubtedly function in redox reactions, possibly where thioredoxin-like proteins transfer electron equivalents for thioredoxin-dependent ArsC reduction reactions [38].

### 3.10. The ArsA Superfamily

A Conserved Domain Search (CD-Search) of the NCBI protein database using the *E. coli* ArsA as the query sequence brought up a variety of ArsA-like ATPases with known associations. The AAA+ superfamily includes many proteins of different presumed physiological functions. Characterization of the AAA+ superfamily has been reported [39–41]. Our tree examines the AAA+ superfamily homologues of ArsA that are most closely related to the ArsA of *E. coli*. Based on NCBI descriptions of the conserved domains, (1) the FleN-like ATPase regulates motility by interacting with FleQ. (2) the ParA enzyme *Caulobacter crescentus* functions in chromosome segregation; when ADP binds, the protein interacts with single-stranded (ss) DNA, but when ATP is bound, ParB dissociates from its DNA binding sites. (3) CooC is a protein involved in maturation of the nickel center of carbon monoxide dehydrogenase. (4) The Fer4 or NifH protein uses ATP to facilitate the transfer of electrons to N_2_ or partially reduced forms of N_2_ (HN=NH and H_2_N–NH_2_). NifH (component II) hydrolyzes ATP, energizing the transfer of electrons through an Fe_4_–S_4_ cluster to the other subunits within the nitrogenase complex. (5) MinD is a membrane-associated ATPase that regulates MinC and MinE, which function in the formation of the bacterial midcell septum. (6) BchL-like and ChlL proteins catalyze reductive formation of chlorophyllide. (7) An MRP-like ArsA homologue may be a component of an Na^+^/H^+^ antiporter complex. (8) CbiA catalyzes the ATP-dependent amidation of various side chains of hydrogenobyrinic acid or cobyrinic acid a,c diamide in the synthesis of vitamin B_12_. (9) DnaC is a DNA replication protein. (10) FlhG is an antiactivator of FleN. (11) Soj is an ATPase involved in chromosome partitioning in *B. subtilis* with homologues of the same function in many bacteria. (12) MipZ is an ATPase that forms a complex with the chromosome partitioning ParB and regulates FtsZ ring formation. (13) HslU is the ATPase component of the HslUV ATP-regulated protease/chaperone complex.

**Figure 4:**
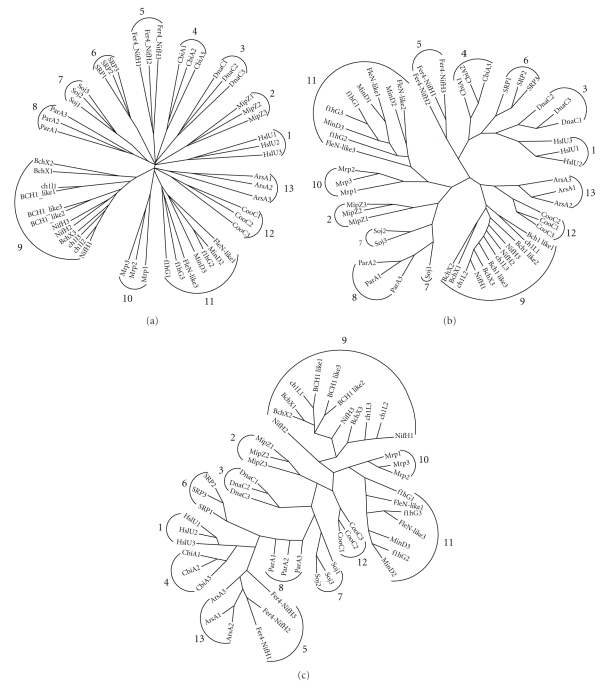
(a) CLUSTALX multiple alignment showing the relationships of the representative proteins to each other. Representative proteins from each family were selected following an NCBI BLASTP search, selecting sequences with 30%–50% identity and an *e*-value less than *e* ^−60^. (b) SuperfamilyTree created with representative proteins using the SFT1 program [22] without a multiple alignment and using TreeView [24]. (c) Representative proteins were analyzed, and the tree was generated using ProtPars [25]. Both CLUSTALX and SFT1 use neighbor-joining algorithms to generate the trees while ProtPars uses parsimony. These trees show the evolutionary relationships of ArsA homologues to each other. Numbers are assigned in (a) and retained in (b) and (c).

We investigated the relationships between the various ArsA homologues with CLUSTALX (Figure 4(a)), SuperfamilyTree1 (SFT1) (Figure 4(b)), and ProtPars (Figure 4(c)). CLUSTALX and SFT1 utilize neighbor-joining algorithms while ProtPars relies on parsimony. CLUSTALX and ProtPars base branching patterns on multiple alignments, but SFT1 uses BLAST bit scores [22]. Several patterns emerge when comparing the predicted evolutionary pathways taken by these families. In all three trees we find that Clusters 10 (Mrp) and 11 (MinD, FlhG, and FleN-like) are together, Clusters 1 (HslU), 4 (CbiA), and 5 (Fer4_NifH) are together, and Clusters 7 (Soj) and 8 (ParA) are together. Clusters 9 (BchX, ChlL, NifH, and Bch1-like), 10 (MRP), 11 (MinD, FlhG, and FleN-like), and 12 (CooC) are found together in trees A and C. Clusters 1 (HslU), 3 (DnaC), 4 (CbiA), and 5 (Fer4_NifH) are found together in trees A and B. Finally, Clusters 12 (CooC) and 13 (ArsA) are found together in trees A and B. These results provide information about the relative phylogenetic distances for these AAA+ ATPases, thus suggesting their functional/mechanistic relationships.

### 3.11. Orthologous Relationships Based on Comparison with 16S and 18S rRNA Phylogeny

**Figure 5:**
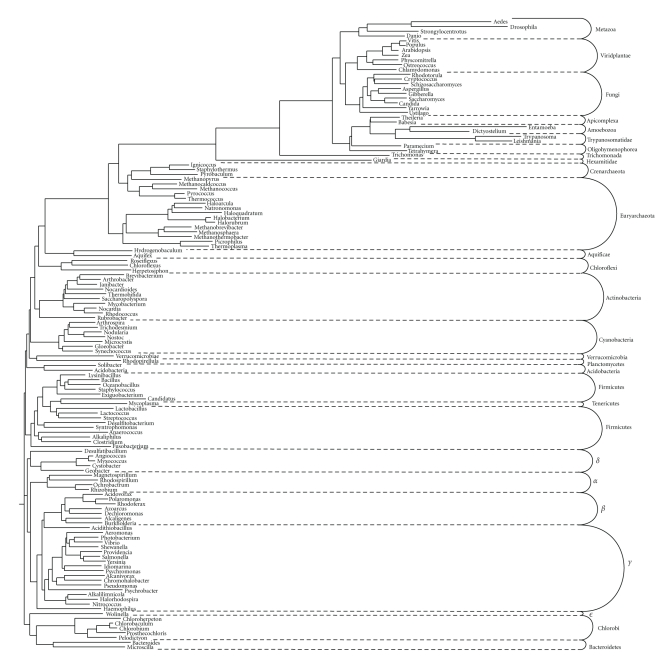
Phylogenetic tree of 16S and 18S rRNA nucleotide sequences of genera represented in this study. The methodology was as described in Figure 1. Sequences were derived from the NCBI nucleotide database. Clusters correlate with organismal type.

The ribosomal RNA (rRNA) tree (Figure 5) for the organisms included in this study exhibits clustering patterns pretty much as expected with the different phyla grouping separately. Of note, the one representative *ε*-proteobacterial RNA clusters loosely with the Bacteroidetes homologues instead of the large proteobacterial RNA cluster.

As indicated in Table 1, Cluster 1 of Figure 4 includes proteins derived from proteobacteria and other phyla, each clustering together, but with many exceptions. For example, Bth1 (Bacteroidetes) is sandwiched in between Firmicute homologues. Clustering together in the following order are Gur1 (*δ*-Proteobacterium), Bph1 (*β*-Proteobacterium), Mma1, and Rru1 (*α*-Proteobacteria). Additionally, we find two proteins from Verrucomicrobia and Planctomycetes, respectively, clustering together with these proteobacterial proteins. Also, among a group of proteobacterial proteins, we find one homologue from Chlorobi and three from Actinobacteria. Euryarchaeata proteins cluster separately at the base of Cluster 1 as well as in Cluster 2. These results clearly demonstrate that there has been extensive horizontal *arsA* gene transfer during the evolution of these organisms.

Cluster 3 proteins are derived exclusively from eukaryotes. However, many different phyla are represented. Progressing in the clockwise direction, the first two proteins, which group together, are from Trichomonada species. Then we find a cluster of fungal proteins with two deeply rooted members, Cal1 and Sce1, possible examples of horizontal gene transfer. Tad1 is a metazoan protein which clusters tightly together with four-plant proteins. This also appears to be an example of horizontal gene transfer since all other Metazoan proteins cluster separately. Remaining proteins in Cluster 3 are relatively distantly related to each other and are derived from four distinct phyla. Since proteins from each phylum cluster together, it is reasonable to suggest that these were inherited by vertical descent.

Cluster 4 consists of proteins derived from Cyanobacteria with one exception, a *δ*-proteobacterial protein. This last protein is more distantly related to the cyanobacterial proteins than they are to each other. Further, the protein and rRNA trees for Cyanobacteria are in agreement. Therefore, the results are consistent with orthology for all of these proteins. It should be noted that genome context analyses indicated that these proteins are associated with gas vesicle biogenesis.

Cluster 5 consists exclusively of plant and algal proteins. While much of the evidence suggests orthology, it should be noted that *Arabidopsis*, *Vitis*, and *Zea* all have two paralogues which, however, do not exhibit orthologous relationships between them. It seems likely that these proteins arose by more than one gene duplication event. Cluster 6 consists only of two distantly related proteins from two different phyla, while Cluster 7 consists of archaeal proteins with phylogenetic relationships consistent with orthology. These proteins may function together with CstA in carbon stress responses.

Cluster 8 includes proteins from eight different bacterial phyla. Moreover, the Chloroflexi and Chlorobi proteins each fall into three distinct subclusters. The two *δ*-proteobacterial proteins are also distantly related. The results are consistent either with horizontal gene transfer or with early gene duplication events.

Cluster 9 includes proteins from four different bacterial and archaeal phyla. These clearly do not exhibit orthology, but as for Cluster 8, the basis for this observation is not clear. Cluster 10 consists of proteins only from Actinobacteria, and with only one protein derived from any one genus; the results are fully consistent with orthology (compare Figures 1 and 5). Cluster 11 consists of only four proteins derived from three different phyla. Orthology seems unlikely. Clusters 12 and 13 each consists of two distantly related proteins from two different phyla. We are hesitant to predict orthology for these proteins. Cluster 14 consists of six proteins. Two distant members derive from Crenarchaeota, and four close members derive from Euryarchaeota. The results are consistent with orthology. Cluster 15 consists of four proteins two from archaea and two from bacteria. Surprisingly, each of the bacterial proteins clusters together with an archaeal protein, and the phylogenetic distance is the same for these two pairs. The basis for these relationships is unknown, but horizontal transfer probably played a role.

The three proteins in Cluster 16 exhibit relationships potentially consistent with orthology. Cluster 17 consists of twelve proteins from three bacterial phyla and one archaeal phylum. The *γ*-proteobacterial proteins fall into three distinct clusters, inconsistent with orthology. The four archaeal proteins cluster separately from the bacterial proteins, and form relationships suggesting that only the Haloarcular homologue may not be orthologous to the others. It seems unlikely that the members of Cluster 18 are orthologous.

In summary, most of the 18 clusters consist of proteins that are not likely to be orthologous although a few exceptions have been noted. While horizontal gene transfer appears to account for much of this divergence, we consider it equally likely that early gene duplication events, giving rise to multiple paralogues, occurred within this protein family.

### 3.12. Conserved Motifs in ArsA Homologues

**Figure 6:**
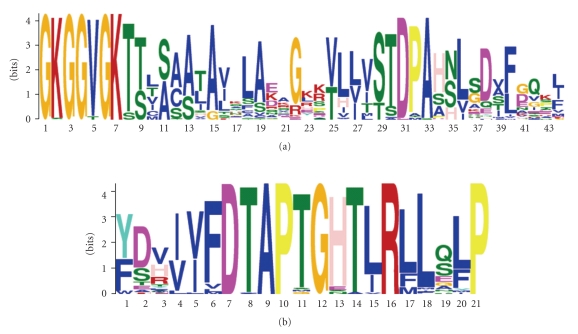
MEME [30] sequence logos illustrating the conserved residues of (a) motif 1 and (b) motif 2 found in ArsA homologues. The size of the letters indicates amino acid conservation, with larger letters representing more conserved residues.

The MEME program [30] was used to examine the occurrence of conserved motifs within the 112 ArsA homologues included in the training set, 80 single domain and 32 double domain proteins. The two most conserved motifs obtained with this program are shown in Figure 6. The best conserved motif (motif 1 in Figure 6) includes the Rossmann fold motif (GXGXXG) which is fully conserved at the beginning of this motif. The sequence of this fully conserved motif (positions 1–7) plus the well-conserved adjacent residues at positions 8–13 is GKGGVGK(T/S)_2_X(S/A)(A/C/S)(A/S) [alternative residues at a single position are in parentheses]. Additionally, at positions 29–33, we found another well-conserved submotif (S/T)(T/S)DPA where the only substitutions are in the P which is replaced by an A, T, or M in just four of the fifty homologues included in this analysis. As expected, the single domain proteins had a single such motif while the double domain proteins had two such motifs which differed from each other in no significant way. 

The second best conserved motif is the 12-residue motif (DTAPTGHTIRLL), called the DTAP motif [9]. The DTAP motif appears to be a conformationally flexible region which facilitates the interconversion of the ATP/ADP substrate/product binding site(s). This exceptionally well-conserved motif corresponds precisely to that reported by Rensing et al. [9]. Additionally, following the Rossmann fold motif, an SXD motif is observed with the S and D almost fully conserved. Finally, towards the C-terminus of these proteins is a well-conserved N residue that is fully conserved in the single domain proteins (see below).

Three cysteine residues have been shown to be important for the activity of the *E. coli* ArsA protein, C113, C172, and C422 [9]. The conservation of these cysteines was examined separately in multiple alignments of both the double and the single domain proteins. Double domain proteins can be found in Clusters 1, 4, and 6, while all remaining clusters include proteins with only a single domain. None of these three cysteine residues were conserved in the single domain proteins. In all Cluster 1 proteins, the C is fully conserved with a single exception: C172 is replaced by an N in Sty1. However, in Clusters 4 and 6, none of these cysteines are well conserved. In fact, a W is found in all but one (Gur2) of the Cluster 4 proteins, where an F can be found, for C113, while an L and a V replace the C residue in the two cluster 6 proteins. C172 is replaced by an S or T in Cluster 4 proteins except for Gur2 where an R is found. V and K residues replace this C in the two Cluster 6 proteins. C422 is fully conserved in Cluster 1 proteins, but in Cluster 4 homologues, an aliphatic hydrophobic residue always occurs. Finally, in the two Cluster 6 proteins a G and an L occur. It seems clear from this analysis that the mechanism of action of Cluster 1 homologues differs substantially from that of all other proteins in the ArsA tree (Figure 1).

The single domain proteins were separately aligned, and they were found to have a well-conserved Rossmann fold motif GKGG(V/T)GK(T/S). The DTAP motif could also be recognized, but it was less well conserved than for the double domain proteins. Additionally, the three well-conserved cysteines present in the double domain proteins and essential for activity for the *E. coli* protein were not conserved in any of the single domain proteins. However, two regions in these single domain proteins proved to be well conserved. These were the SXD motif in the beginning of the proteins, where the S is fully conserved and the D can be substituted only by N, and the fully conserved C-terminal N residue mentioned above. Thus, it is clear that the mechanisms of action must be quite different, but these differences distribute largely according to phylogenetic clusters.

## 4. Discussion

The apparent functional promiscuity of ArsA homologues is worthy of note. Our phylogenetic analyses have provided evidence for the coevolution of different sets of ArsA homologues with (1) ArsB, ArsP, and frequently ArsD, (2) CstA and CstX, and (3) gas vesicle biogenesis proteins. However, ArsA and Acr3 homologues did not coevolve, and the few ArsH homologues identified also appeared to have evolved independently of ArsA. Nevertheless, we have demonstrated that *arsB*, *arsP, arsD*, and *acr3* often occur within the same operons as *arsA* homologues; *cstA*, *cstX*, and *cstY* are also found in operons with *arsA* homologues, and CstA “looks” like a permease. Coevolution and coclustering of the encoding genes suggest common functions and mutual interactions.

To support the hypothesis that ArsA homologues function in conjunction with these various permeases, we have examined instances in which an *arsA* homologue is found in an operon containing one of the four putative transporters mentioned above in the absence of the other three. We have identified nine such instances for Acr3, eleven for ArsP, ten for ArsB, and 26 for CstA. Strikingly, none of the arsenic resistance genes are found in operons with carbon starvation genes. The converse is true as well, clearly suggesting a function in arsenic resistance for the former gene sets, but not for the latter.

Based on phylogenetic trees, we suggest that ArsA has coevolved with the above proteins with the notable exception of Acr3. We therefore hypothesize that ArsA homologues function not only with ArsB but also, in specific instances, with ArsP and CstA. Analysis of potential coevolving protein pairs, for proteins not present within the same operons or gene clusters, revealed a few cases of possible coevolution, but in many cases, coevolution seemed impossible.

It should be noted that although Acr3 may not have coevolved with ArsA homologues, *acr3* genes are found nine times within operons also containing *arsA* homologues but lacking *arsB* and *arsP* genes anywhere in the genomes. There are 23 instances in which *acr3* genes are found with an *arsA* homologue in an operon without an *arsB*. Thus, ArsA homologues may either function with an unidentified protein not found in the operon or with the cotranscribed *acr3* homologue. A recent study has proposed, based upon biochemical analyses, that ArsA homologues do indeed function with Acr3 [17]. A cloned *A. metalliredigens arsa1* operon, contains two single domain *arsA* homologues and an *acr3* homologue. When transferred to *E. coli*, increased resistance to arsenite was observed when the *arsA* and *acr3* homologues were expressed together. In contrast, no such increased resistance was observed when the *acr3* homologue was expressed alone [17]. Given the widespread colocalization of *arsA* and *acr3* homologues and the recent biochemical analyses of Fu et al. [17], we agree that ArsA is likely to energize Acr3-mediated arsenite efflux.

The case of Outer Membrane Pore-Forming Factors (OMFs) is particularly relevant to this discussion. OMF proteins function with various cytoplasmic membrane transport systems but did not coevolve with these systems [34]. An explanation might be promiscuity with respect to the protein-protein interactions required for function. It is known, for example, that a single OMF (e.g., TolC of *E. coli*) can function with several different transporters, although the cofunctioning Membrane Fusion Proteins (MFPs) do not (see TCDB). Thus, proteins that exhibit a lack of coevolution can still function together, but the physical association may be less stringent.

Conserved motifs in ArsA homologues were examined using the MEME program [30]. Concerning the internally duplicated ArsA homologues of phylogenetic clusters 1, 4, and 6, the Rossmann fold and surrounding regions proved to be almost identical, and consequently only the motif for the first halves, together with all single domain homologues, was presented. The Rossmann fold was fully conserved in virtually all homologues, suggesting that none of the proteins included in our study were the products of pseudogenes. Additionally, the DTAP domain, thought to facilitate the interconversion of the ATP/ADP substrate/product binding site(s), also proved to be remarkably well conserved among all of the homologues examined. In fact, conservation in both motifs was more extensive than previously recognized, suggesting that additional residues will prove to be crucial for function. It is also worthy of note that the exceptionally well-conserved N-terminal SXD and C-terminal N residues are also present in the two domain proteins, as well as all homologues, again suggesting that these residues play a role in the basic mechanism used by these proteins.

The remarkable conservation of function noted above contrasts with the lack of conservation of the three cysteine residues, which in the *E. coli* ArsA protein have been shown to be essential for function [9]. These residues were fully conserved in Cluster 1, which includes this double domain *E. coli* protein. In fact, all members of this cluster appear to have the same general function and catalyze ATP hydrolysis, coupled to transport by the same mechanism. Of even greater interest is the conclusion that none of the other homologues can function by the same mechanism since they lack these cysteines. These analyses thus indicate not only that the functions of these proteins will differ, but also that their mechanisms of action will differ.

We have found that some homologues of *arsA* are found in operons encoding gas vesicle biogenesis proteins. It can be assumed that the ATPase functions to energize a biogenic step. Since GvpN also appears to be an ATPase, it seems reasonable to propose that these two proteins function to energize distinct but related functions. The Hsp20 protein, also often present in gas vesicle gene clusters, presumably plays a chaperone role in protein complex formation.

By examining the ArsA homologue phylogenetic tree, we found that genes encoding Cluster 1 homologues are associated with *arsB*, *arsP*, and *acr3*, Cluster 4 and 6 homologues are encoded together with gas vesicle biogenesis proteins, and cluster 14–18 homologues are found with carbon starvation genes *cstA*, *cstX*, and sometimes, *cstY*. CstA possibly functions in peptide uptake [42]; CstX might couple CstA-catalyzed transport to ArsA-dependent ATP hydrolysis and is never found encoded in an operon without *cstA*. This fact strongly suggests a close functional and physical relationship between these two gene products. CstY is a protein found in five archaea, encoded within operons together with *cstA* and *cstX*. CstY might well provide a nonessential auxiliary function. Unlike the *ars* operons, the gas vesicle and carbon starvation operons are probably not controlled by arsenite concentrations because they do not contain associated *arsR* genes.

ArsA homologues associated with arsenic resistance and gas vesicle biogenesis have two domains and are ~600 aas long. In contrast, ArsA homologues associated with carbon starvation genes have one domain and are  ~300 aas long. Why the internally duplicated ArsAs are always associated with arsenic resistance or *gvp* genes while those that function with carbon starvation proteins possess just one ArsA domain is unknown. The latter ArsAs may function as homodimers. We have also found 60 new instances of ArsP homologues based on their association with other *ars* operon determinants. Although the function of ArsP homologues is unknown, they might play a backup role in As(III) export or act on a related substrate.

We have identified three putative oxidoreduction genes present in *ars* operons. They encode homologues of CcdA, thioredoxin, and redox-active disulfide protein 2 which may function with ArsC, the As(V) reductase. Thioredoxin is likely to provide electrons to ArsC, and CcdA might transfer electrons across the membrane as does DsbD. However, not all DsbD homologues appear to catalyze transmembrane electron flow. A function in transmembrane electron flow seems less likely since ArsC is cytoplasmic. Clearly the reported studies open up many avenues of molecular genetic inquiry.

## Supplementary Material

Figure S1: Dendogram of the phylogenetic tree shown in Fig. 1. Proteins are arranged according to cluster and order within the cluster as seen in Fig. 1.
Figure S2: Phylogenetic trees showing the coevolution of (A) ArsA and (B) ArsB homologues, (C) ArsA and (D) Acr3 homologues, and (E) ArsA and (F) ArsP homologues, encoded within the same genomes. For these trees, we use the following system of nomenclature to better designate the added protein pairs not found within the same operons. The first digit indicates separate organisms with the same three letter code (ex: Aaa1, Aaa2), the second digit indicates paralogues, if present (ex: Aaa1-1, Aaa1-2). If the transporter is located within the same genome, but not the same operon as the*arsA*, the letter E, standing for elsewhere, follows the name (ex: Aaa1-3E).
Figure S3: Phylogenetic trees showing the coevolution of (A) ArsA and (B) ArsD homologues encoded within the same operon. The methodology was as described in Fig. 1.

## Figures and Tables

**Table 1:** ArsA homologues used to generate the phylogenetic tree. From left to right, the proteins are characterized according to cluster and abbreviation, organismal source, protein size, organismal type, and gi number. Within each cluster, proteins are presented according to position going clockwise around the tree.

Cluster and protein abbreviations	Organismal source	Protein size (no. of aas)	Organismal type	GI no.
Cluster 1				
Sep1	Staphylococcus epidermidis RP62A	565	Firmicutes	57865830
Esp2	Exiguobacterium sp. AT1b	585	Firmicutes	187604364
Lsp2	Lysinibacillus sphaericus C3-41	586	Firmicutes	169828142
Lpl1	Lactobacillus plantarum WCFS1	576	Firmicutes	54307196
Sdy1	Streptococcus dysgalactiae subsp. equisimilis	580	Firmicutes	157419736
Lla1	Lactococcus lactis subsp. cremoris MG1363	571	Firmicutes	125624073
Hin1	Haemophilus influenzae PittHH	597	*γ*	145636206
Ahy1	Anaerococcus hydrogenalis DSM 7454	580	Firmicutes	212696774
Bth1	Bacteroides thetaiotaomicron VPI-5482	570	Bacteroidetes	29345526
Bco1	Bacillus coagulans 36D1	590	Firmicutes	124520553
Swo1	Syntrophomonas wolfei subsp. wolfei str. Goettingen	583	Firmicutes	114566470
Cte6	Clostridium tetani E88	589	Firmicutes	28211528
Cph2	Clostridium phytofermentans ISDg	582	Firmicutes	160880289
Dha1	Desulfitobacterium hafniense Y51	598	Firmicutes	89897344
Aor1	Alkaliphilus oremlandii OhILAs	582	Firmicutes	158320145
Cbe1	Clostridium beijerinckii NCIMB 8052	582	Firmicutes	150016979
Bse1	Bacillus selenitireducens MLS10	587	Firmicutes	163763208
Bce2	Bacillus cereus ATCC 10987	586	Firmicutes	44004495
Bsp2	Bacillus sp. SG-1	594	Firmicutes	149180106
Gur1	Geobacter uraniireducens Rf4	583	*δ*	148263449
Bph1	Burkholderia phytofirmans PsJN	584	*β*	187925677
Mma1	Magnetospirillum magnetotacticum MS-1	574	*α*	46201533
Rru1	Rhodospirillum rubrum ATCC 11170	571	*α*	83592783
Bvi1	Burkholderia vietnamiensis G4	587	*β*	134293367
Asp4	Azoarcus sp. BH72	582	*β*	119898647
Rba1	Rhodopirellula baltica SH 1	593	Planctomycetes	32475953
Aeh2	Alkalilimnicola ehrlichei MLHE-1	571	*γ*	114321857
Orf3	delta proteobacterium MLMS-1	592	*δ*	94266138
Vba1	Verrucomicrobiae bacterium DG1235	584	Verrucomicrobia	198257295
Vha1	Vibrio harveyi ATCC BAA-1116	582	*γ*	156973785
Sty1	Salmonella typhimurium	585	*γ*	32470159
Ahy2	Aeromonas hydrophila subsp. hydrophila ATCC 7966	599	*γ*	117618702
Ppr1	Photobacterium profundum SS9	590	*γ*	54308735
Psp1	Psychromonas sp. CNPT3	602	*γ*	90408036
Cfe1	Chlorobium ferrooxidans DSM 13031	591	Chlorobi	110598505
Psp2	Pseudomonas sp. TS44	585	*γ*	170026534
Rfe1	Rhodoferax ferrireducens T118	589	*β*	89902424
Dar1	Dechloromonas aromatica RCB	590	*β*	71908250
Afa1	Alcaligenes faecalis	591	*β*	42741719
Pae1	Pseudomonas aeruginosa	588	*γ*	187939925
Sen1	Salmonella enterica subsp. enterica serovar Saintpaul str. SARA29	586	*γ*	167553198
Aar1	Aromatoleum aromaticum EbN1	592	*β*	56478279
Aca1	Acidithiobacillus caldus	612	*γ*	60686969
Otr1	Ochrobactrum tritici	582	*α*	94483089
Pst2	Providencia stuartii ATCC 25827	594	*γ*	188026271
Ssp2	Shewanella sp. ANA-3	588	*γ*	117920786
Yin1	Yersinia intermedia ATCC 29909	586	*γ*	77979455
Asp1	Arthrobacter sp. FB24	621	Actinobacteria	116668777
Mgi1	Mycobacterium gilvum PYR-GCK	589	Actinobacteria	145222246
Rer1	Rhodococcus erythropolis	585	Actinobacteria	33867198
Rle1	Rhizobium leguminosarum bv. viciae 3841	588	*α*	116254241
Pna1	Polaromonas naphthalenivorans CJ2	600	*β*	121606619
Asp2	Acidovorax sp. JS42	586	*β*	121594853
Hla2	Halorubrum lacusprofundi ATCC 49239	640	Euryarchaeota	153896608
Hsp4	Halobacterium sp. NRC-1	644	Euryarchaeota	10803670

Cluster 2				
Mka1	Methanopyrus kandleri AV19	333	Euryarchaeota	20095116
Mst1	Methanosphaera stadtmanae DSM 3091	328	Euryarchaeota	84488998
Msm1	Methanobrevibacter smithii ATCC 35061	340	Euryarchaeota	148643230
Mth1	Methanothermobacter thermautotrophicus str. Delta H	324	Euryarchaeota	15679508
Mja1	Methanocaldococcus jannaschii DSM 2661	349	Euryarchaeota	15669329
Mma2	Methanococcus maripaludis S2	345	Euryarchaeota	45357726
Mae2	Methanococcus aeolicus Nankai-3	341	Euryarchaeota	150401428

Cluster 3				
Tva1	Trichomonas vaginalis G3	297	Trichomonada	123416597
Tva2	Trichomonas vaginalis G3	292	Trichomonada	123451254
Yli1	Yarrowia lipolytica CLIB122	327	Fungi	50554649
Gze1	Gibberella zeae PH-1	341	Fungi	46136751
Ani1	Aspergillus nidulans FGSC A4	340	Fungi	67524903
Spo1	Schizosaccharomyces pombe	329	Fungi	19115182
Rgl1	Rhodotorula glutinis	339	Fungi	183396512
Uma1	Ustilago maydis 521	332	Fungi	71019509
Cci1	Coprinopsis cinerea okayama7#130	326	Fungi	169843560
Cne1	Cryptococcus neoformans var. neoformans JEC21	325	Fungi	58260906
Cal1	Candida albicans SC5314	350	Fungi	68468811
Sce1	Saccharomyces cerevisiae	354	Fungi	51013779
Tad1	Trichoplax adhaerens	339	Metazoa	196008131
Ppa2	Physcomitrella patens subsp. patens	365	Viridiplantae	168012492
Obr1	Oryza brachyantha	364	Viridiplantae	110430665
Ath2	Arabidopsis thaliana	345	Viridiplantae	8570442
Psi1	Picea sitchensis	374	Viridiplantae	116784166
Ddi1	Dictyostelium discoideum AX4	329	Amoebozoa	66800287
Api1	Acyrthosiphon pisum	339	Metazoa	193582608
Dre1	Danio rerio	341	Metazoa	50539666
Nve1	Nematostella vectensis	334	Metazoa	156398556
Spu1	Strongylocentrotus purpuratus	346	Metazoa	72050675
Dan1	Drosophila ananassae	336	Metazoa	194755601
Nvi1	Nasonia vitripennis	344	Metazoa	156537421
Aae3	Aedes aegypti	341	Metazoa	157128460
Tca1	Tribolium castaneum	330	Metazoa	91081505
Cel1	Caenorhabditis elegans	342	Metazoa	17557003
Bma1	Brugia malayi	344	Metazoa	170590260
Pte1	Paramecium tetraurelia strain d4-2	325	Oligohymenophorea	145545770
Tth1	Tetrahymena thermophila SB210	349	Oligohymenophorea	118401519
Tcr1	Trypanosoma cruzi strain CL Brener	359	Trypanosomatidae	71401129
Lma1	Leishmania major strain Friedlin	409	Trypanosomatidae	157865666
Tpa1	Theileria parva strain Muguga	361	Apicomplexa	71027033
Bbo1	Babesia bovis T2Bo	358	Apicomplexa	156082722
Pbe1	Plasmodium berghei str. ANKA	379	Apicomplexa	68071753
Cmu1	Cryptosporidium muris RN66	390	Apicomplexa	209879305
Cpa1	Cryptosporidium parvum Iowa II	366	Apicomplexa	126654216
Ehi1	Entamoeba histolytica HM-1:IMSS	327	Amoebozoa	67466277
Gla1	Giardia lamblia ATCC 50803	354	Hexamitidae	159119999

Cluster 4				
Gur2	Geobacter uraniireducens Rf4	637	*δ*	148264869
Ssp1	Synechococcus sp. JA-3-3Ab	684	Cyanobacteria	86605793
Mae1	Microcystis aeruginosa PCC 7806	633	Cyanobacteria	159025965
Nsp2	Nostoc sp. PCC 7120	635	Cyanobacteria	17229736
Nsp3	Nodularia spumigena CCY 9414	617	Cyanobacteria	119512417
Ama1	Arthrospira maxima CS-328	637	Cyanobacteria	209527482
Ter2	Trichodesmium erythraeum IMS101	626	Cyanobacteria	113475961
Npu1	Nostoc punctiforme PCC 73102	623	Cyanobacteria	186682498

Cluster 5				
Zma2	Zea mays	374	Viridiplantae	195625344
Ath3	Arabidopsis thaliana	391	Viridiplantae	30697424
Vvi1	Vitis vinifera	422	Viridiplantae	147852937
Ppa1	Physcomitrella patens subsp. patens	359	Viridiplantae	168024699
Zma1	Zea mays	394	Viridiplantae	195645964
Ath1	Arabidopsis thaliana	411	Viridiplantae	30681260
Vvi2	Vitis vinifera	409	Viridiplantae	157343988
Ptr1	Populus trichocarpa	407	Viridiplantae	118487322
Cre1	Chlamydomonas reinhardtii	513	Viridiplantae	159488560
Olu1	Ostreococcus lucimarinus CCE9901	330	Viridiplantae	145350244

Cluster 6				
Aba1	Acidobacteria bacterium Ellin345	634	Acidobacteria	94969437
Mxa1	Myxococcus xanthus DK 1622	655	*δ*	108758691

Cluster 7				
Hwa1	Haloquadratum walsbyi DSM 16790	312	Euryarchaeota	110668350
Orf2	uncultured prokaryote 2E01B	314	none	85372676
Nph1	Natronomonas pharaonis DSM 2160	317	Euryarchaeota	76801342
Nph2	Natronomonas pharaonis DSM 2160	370	Euryarchaeota	76801234
Hsp2	Halobacterium sp. NRC-1	347	Euryarchaeota	15789625
Hla3	Halorubrum lacusprofundi ATCC 49239	392	Euryarchaeota	153895127

Cluster 8				
Cth5	Chloroherpeton thalassium ATCC 35110	405	Chlorobi	193215201
Cau4	Chloroflexus aurantiacus J-10-fl	399	Chloroflexi	163845724
Cte1	Chlorobium tepidum TLS	395	Chlorobi	21673808
Cth2	Chloroherpeton thalassium ATCC 35110	405	Chlorobi	193214297
Pae2	Prosthecochloris aestuarii DSM 271	404	Chlorobi	194332962
Cte3	Chlorobium tepidum TLS	398	Chlorobi	21672957
Plu2	Pelodictyon luteolum DSM 273	406	Chlorobi	78185960
Cte2	Chlorobium tepidum TLS	405	Chlorobi	21674757
Cth4	Chloroherpeton thalassium ATCC 35110	402	Chlorobi	193214011
Cau1	Chloroflexus aurantiacus J-10-fl	390	Chloroflexi	163846065
Dau1	Candidatus Desulforudis audaxviator MP104C	397	Firmicutes	169830523
Ame1	Alkaliphilus metalliredigens QYMF	391	Firmicutes	150389286
Bce1	Bacillus cereus ATCC 10987	392	Firmicutes	42779427
Rxy1	Rubrobacter xylanophilus DSM 9941	394	Actinobacteria	108804153
Pae3	Prosthecochloris aestuarii DSM 271	395	Chlorobi	194333066
Cte4	Chlorobium tepidum TLS	394	Chlorobi	21674880
Cli1	Chlorobium limicola DSM 245	395	Chlorobi	189347837
Sel1	Synechococcus elongatus PCC 7942	392	Cyanobacteria	81300068
Gvi1	Gloeobacter violaceus PCC 7421	394	Cyanobacteria	37520959
Adi1	Angiococcus disciformis	405	*δ*	53747901
Hsp1	Hydrogenobaculum sp. Y04AAS1	397	Aquificae	195953882
Aae2	Aquifex aeolicus VF5	396	Aquificae	15606091
Hsp5	Hydrogenivirga sp. 128-5-R1-1	393	Aquificae	163783049
Sus1	Solibacter usitatus Ellin6076	395	Acidobacteria	116624985
Dal1	Desulfatibacillum alkenivorans AK-01	397	*δ*	163725491
Hau1	Herpetosiphon aurantiacus ATCC 23779	391	Chloroflexi	159900394
Rsp1	Roseiflexus sp. RS-1	396	Chloroflexi	148655082
Cau3	Chloroflexus aurantiacus J-10-fl	401	Chloroflexi	163848482
Cth3	Chloroherpeton thalassium ATCC 35110	383	Chlorobi	193214006
Cte7	Chlorobium tepidum TLS	384	Chlorobi	21674751

Cluster 9				
Mma4	Microscilla marina ATCC 23134	390	Bacteroidetes	124004922
Pto1	Picrophilus torridus DSM 9790	386	Euryarchaeota	48478270
Tac1	Thermoplasma acidophilum DSM 1728	387	Euryarchaeota	16081559
Cph3	Clostridium phytofermentans ISDg	385	Firmicutes	160879841
Fnu2	Fusobacterium nucleatum subsp. nucleatum ATCC 25586	388	Fusobacteria	19704869
Cph1	Clostridium phytofermentans ISDg	393	Firmicutes	160879840
Fnu1	Fusobacterium nucleatum subsp. nucleatum ATCC 25586	396	Fusobacteria	19704870

Cluster 10				
Nsp1	Nocardioides sp. JS614	410	Actinobacteria	119717335
Jsp1	Janibacter sp. HTCC2649	421	Actinobacteria	84496160
Ser1	Saccharopolyspora erythraea NRRL 2338	400	Actinobacteria	134098280
Nfa1	Nocardia farcinica IFM 10152	436	Actinobacteria	54023683
Mab1	Mycobacterium abscessus	423	Actinobacteria	169629069
Mle1	Mycobacterium leprae TN	415	Actinobacteria	15827412
Msp1	Mycobacterium sp. MCS	421	Actinobacteria	108800245
Msm2	Mycobacterium smegmatis str. MC2 155	425	Actinobacteria	118471162

Cluster 11				
Cth1	Chloroherpeton thalassium ATCC 35110	434	Chlorobi	193214353
Cte5	Chlorobium tepidum TLS	436	Chlorobi	21673187
Cau2	Chloroflexus aurantiacus J-10-fl	407	Chloroflexi	163845728
Cth6	Candidatus Chloracidobacterium thermophilum	420	Acidobacteria	157273534
Cluster 12				
Ter1	Trichodesmium erythraeum IMS101	364	Cyanobacteria	113474690
Bba1	Bdellovibrio bacteriovorus HD100	357	*δ*	42525090

Cluster 13				
Mmy1	Mycoplasma mycoides subsp. mycoides LC str. GM12	304	Tenericutes	188159217
Esp1	Exiguobacterium sp. AT1b	279	Firmicutes	187605346

Cluster 14				
Iho1	Ignicoccus hospitalis KIN4/I	309	Crenarchaeota	156938113
Sma1	Staphylothermus marinus F1	329	Crenarchaeota	126465059
Tba1	Thermococcus barophilus MP	330	Euryarchaeota	197627410
Tko1	Thermococcus kodakarensis KOD1	331	Euryarchaeota	57640929
Pab1	Pyrococcus abyssi GE5	330	Euryarchaeota	14521447
Ton1	Thermococcus onnurineus NA1	330	Euryarchaeota	212224056

Cluster 15				
Par1	Pyrobaculum arsenaticum DSM 13514	334	Crenarchaeota	145591126
Sth2	Symbiobacterium thermophilum IAM 14863	339	Firmicutes	51891819
Par2	Pyrobaculum arsenaticum DSM 13514	327	Crenarchaeota	145591125
Sth1	Symbiobacterium thermophilum IAM 14863	345	Firmicutes	51891818

Cluster 16				
Wsu1	Wolinella succinogenes DSM 1740	313	*ε*	34557874
Aae1	Aquifex aeolicus VF5	299	Aquificae	15605857
Hsp3	Hydrogenivirga sp. 128-5-R1-1	306	Aquificae	163782204

Cluster 17				
Csa1	Chromohalobacter salexigens DSM 3043	313	*γ*	92114127
Bsp1	Bacillus sp. NRRL B-14911	328	Firmicutes	89098562
Bha1	Bacillus halodurans C-125	313	Firmicutes	15614358
Oih1	Oceanobacillus iheyensis HTE831	307	Firmicutes	23098830
Hha1	Halorhodospira halophila SL1	311	*γ*	121998972
Aeh1	Alkalilimnicola ehrlichei MLHE-1	318	*γ*	114319474
Tfu1	Thermobifida fusca YX	301	Actinobacteria	72161795
Nmo1	Nitrococcus mobilis Nb-231	311	*γ*	88811608
Nph3	Natronomonas pharaonis DSM 2160	318	Euryarchaeota	76801557
Hwa2	Haloquadratum walsbyi DSM 16790	327	Euryarchaeota	110667012
Hla1	Halorubrum lacusprofundi ATCC 49239	341	Euryarchaeota	153896540
Hma1	Haloarcula marismortui ATCC 43049	426	Euryarchaeota	55379238

Cluster 18				
Par3	Psychrobacter arcticus 273-4	339	*γ*	71065679
Psp3	Psychrobacter sp. PRwf-1	331	*γ*	148653012
Asp3	Alcanivorax sp. DG881	347	*γ*	196196123
Ilo1	Idiomarina loihiensis L2TR	336	*γ*	56459808
Iba1	Idiomarina baltica OS145	338	*γ*	85712073
Pst1	Pseudomonas stutzeri A1501	335	*γ*	146280770
Bli1	Brevibacterium linens BL2	327	Actinobacteria	62424272
