# Supplementary material for: Functional Promiscuity of Homologues of the Bacterial ArsA ATPases
Source: Int J Microbiol. 2010 Oct 20;2010:187373. doi: 10.1155/2010/187373 (PMC2963123; doi:10.1155/2010/187373)
Supplement: Supplementary file 1 — Figure S1: Dendogram of the phylogenetic tree shown in Fig. 1. Proteins are arranged according to cluster and order within the cluster as seen in Fig. 1. Figure S2: Phylogenetic trees showing the coevolution of (A) ArsA and (B) ArsB homologues, (C) ArsA and (D) Acr3 homologues, and (E) ArsA and (F) ArsP homologues, encoded within the same genomes. For these trees, we use the following system of nomenclature to better designate the added protein pairs not found within the same operons. The first digit indicates separate organisms with the same three letter code (ex: Aaa1, Aaa2), the second digit indicates paralogues, if present (ex: Aaa1-1, Aaa1-2). If the transporter is located within the same genome, but not the same operon as thearsA, the letter E, standing for elsewhere, follows the name (ex: Aaa1-3E). Figure S3: Phylogenetic trees showing the coevolution of (A) ArsA and (B) ArsD homologues encoded within the same operon. The methodology was as described in Fig. 1. [file 187373.f1.pdf]

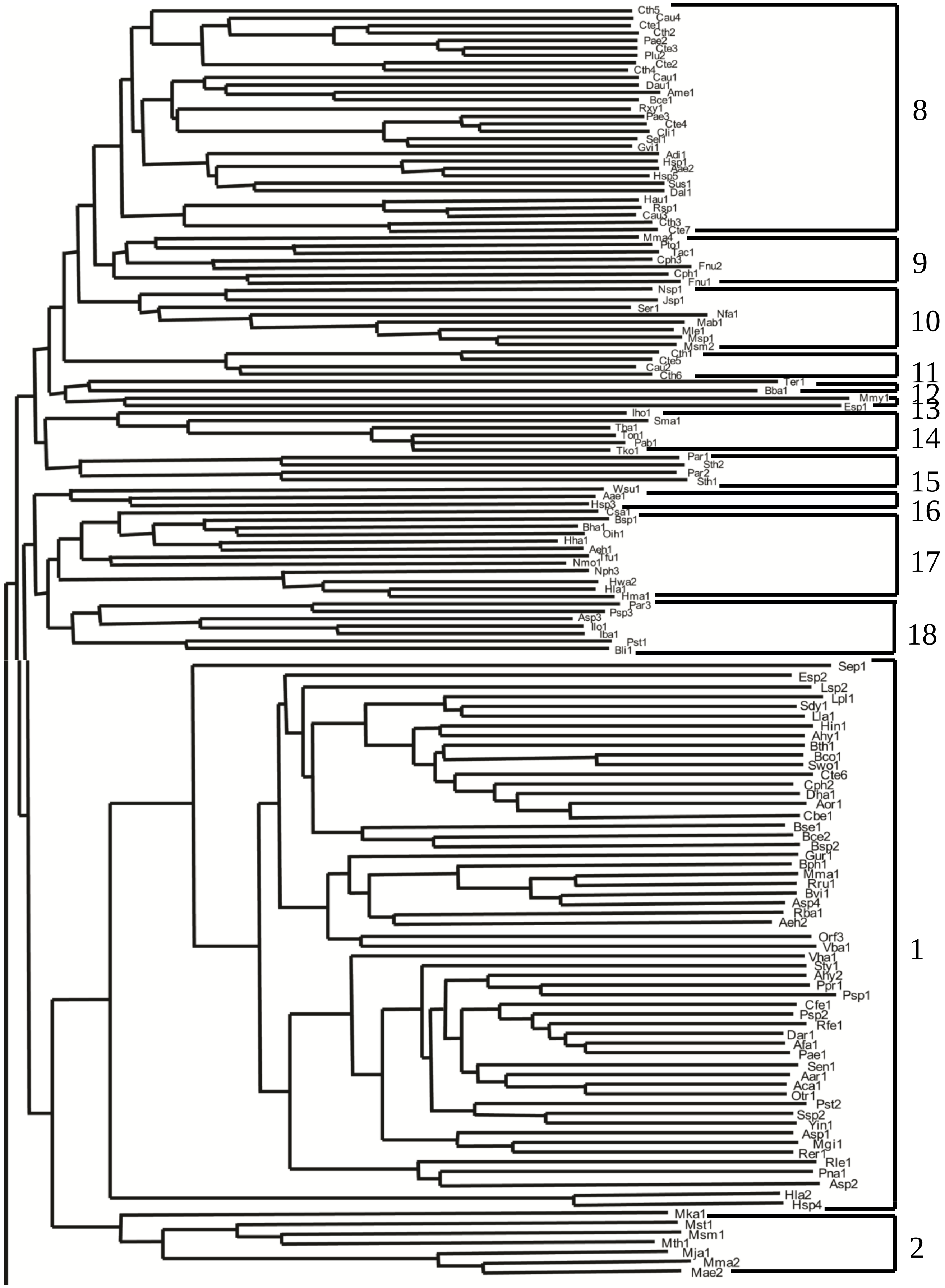
Cth5
Cau4
Cte1
Cth2
Pae2
Cte3
Plu2
Cte2
Cth4
Cau1
Dau1
Ame1
Bce1
Rxy1
Pae3
Cte4
Cli1
Sel1
Gvi1
Adi1
Hsp1
Aae2
Hsp5
Sus1
Dal1
Hau1
Rsp1
Cau3
Cth3
Cte7
8
Mma4
Pto1
Tac1
Cph3
Fnu2
Cph1
Fnu1
9
Nsp1
Jsp1
Ser1
Nfa1
Mab1
Mle1
Msp1
Msm2
10
Cth1
Cte5
Cau2
Cth6
11
Ter1
Bba1
12
Mmy1
Esp1
13
Iho1
Sma1
Tba1
Ton1
Pab1
Tko1
14
Par1
Sth2
Par2
Sth1
15
Wsu1
Aae1
Hsp3
16
Csa1
Bsp1
Bha1
Oih1
Hha1
Aeh1
Tfu1
Nmo1
Nph3
Hwa2
Hla1
Hma1
17
Par3
Psp3
Asp3
Ilo1
Iba1
Pst1
Bli1
18
Sep1
Esp2
Lsp2
Lpl1
Sdy1
Lla1
Hin1
Ahy1
Bth1
Bco1
Swo1
Cte6
Cph2
Dha1
Aor1
Cbe1
Bse1
Bce2
Bsp2
Gur1
Bph1
Mma1
Rru1
Bvi1
Asp4
Rba1
Aeh2
Orf3
Vba1
Vha1
Sty1
Ahy2
Ppr1
Psp1
Cfe1
Psp2
Rfe1
Dar1
Afa1
Pae1
Sen1
Aar1
Aca1
Otr1
Pst2
Ssp2
Yin1
Asp1
Mgi1
Rer1
Rle1
Pna1
Asp2
Hla2
Hsp4
1
Mka1
Mst1
Msm1
Mth1
Mja1
Mma2
Mae2
2

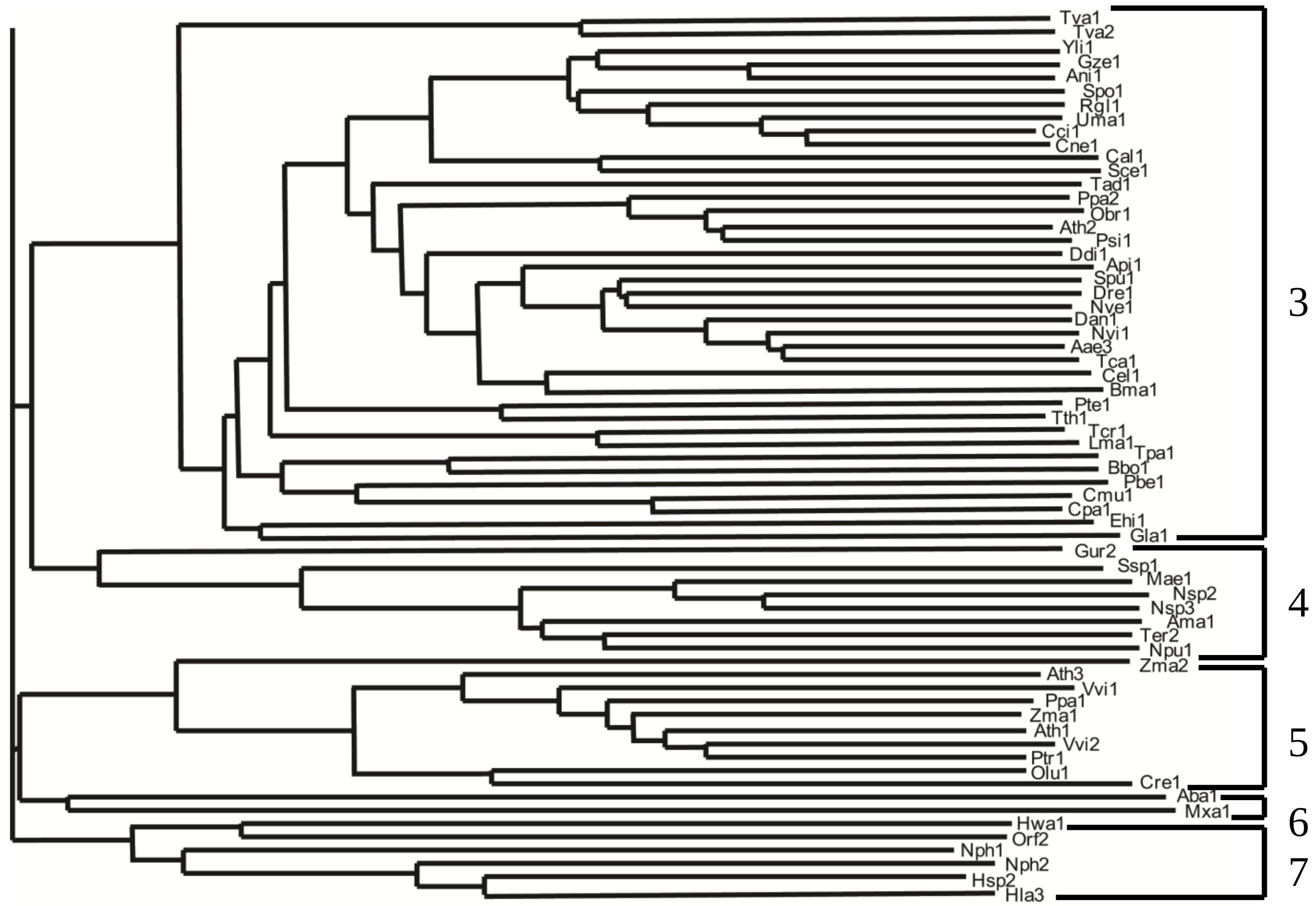

Figure S1: Dendogram of the phylogenetic tree shown in Fig. 1. Proteins are arranged according to cluster and order within the cluster as seen in Fig. 1.

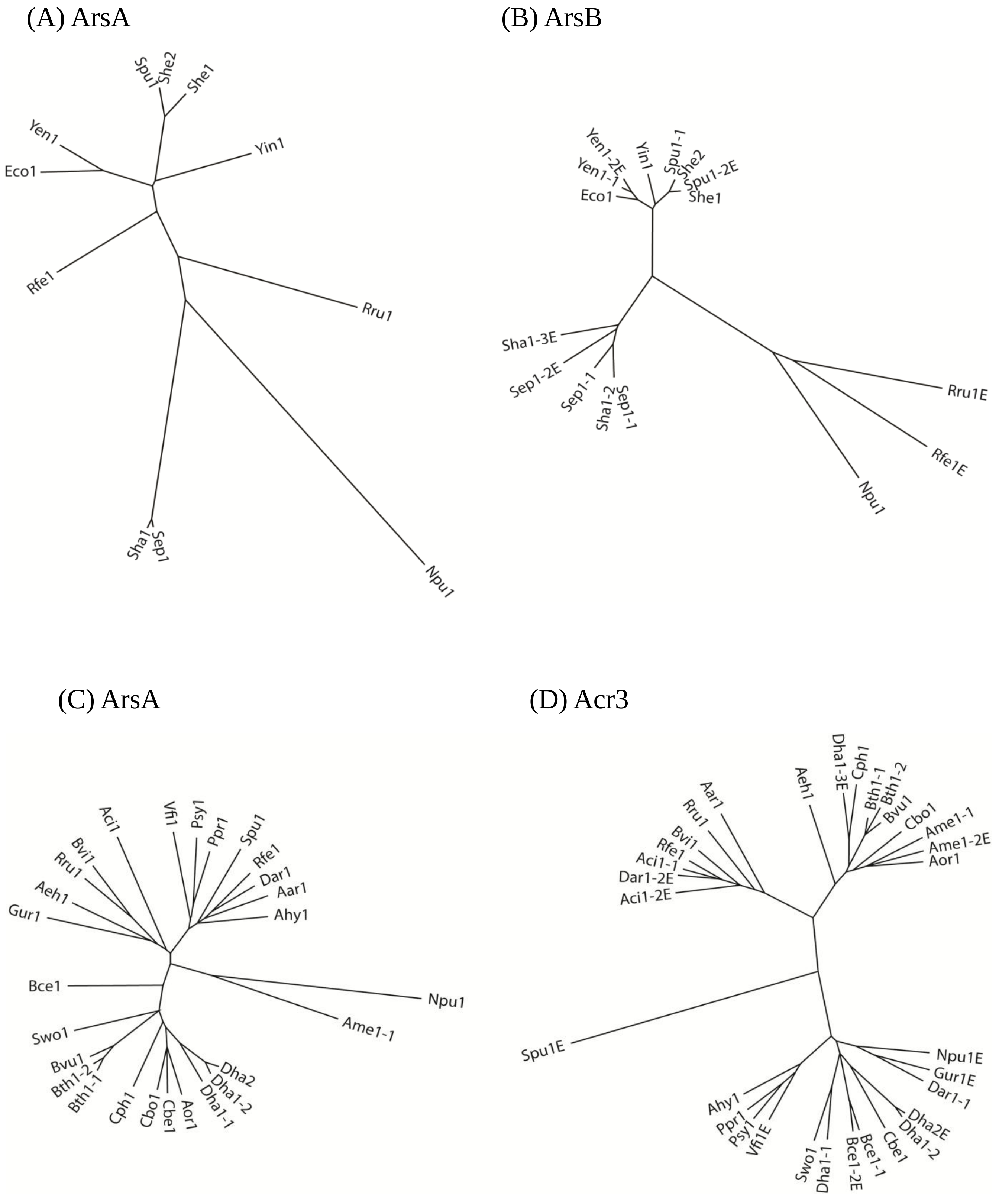
(A) ArsA
Spu1
She2
She1
Yen1
Eco1
Yin1
Rfe1
Rru1
Sha1
Sep1
Npu1
(B) ArsB
Yen1-2E
Yen1-1
Eco1
Yin1
Spu1-1
She2
Spu1-2E
She1
Sha1-3E
Sep1-2E
Sep1-1
Sha1-2
Sep1-1
Rru1E
Rfe1E
Npu1
(C) ArsA
Aci1
Vfi1
Psy1
Ppr1
Spu1
Bvi1
Rru1
Rfe1
Dar1
Aeh1
Aar1
Gur1
Ahy1
Bce1
Npu1
Ame1-1
Swo1
Bvu1
Bth1-2
Bth1-1
Cph1
Cbo1
Cbe1
Aor1
Dha2
Dha1-2
Dha1-1
(D) Acr3
Dha1-3E
Cph1
Aeh1
Bth1-1
Bth1-2
Aar1
Bvu1
Rru1
Cbo1
Bvi1
Ame1-1
Rfe1
Ame1-2E
Aci1-1
Aor1
Dar1-2E
Aci1-2E
Spu1E
Npu1E
Gur1E
Dar1-1
Ahy1
Ppr1
Psy1
Vfi1E
Swo1
Dha1-1
Bce1-2E
Bce1-1
Cbe1
Dha2E
Dha1-2

(E) ArsA (F) ArsP

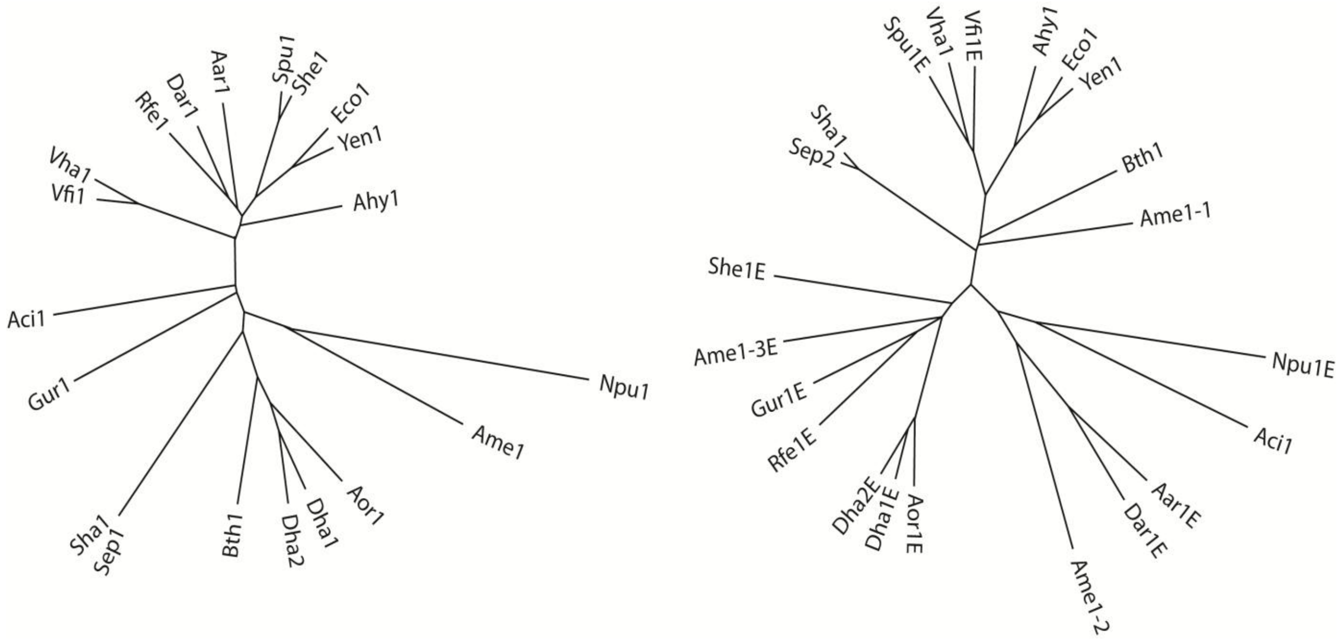

Figure S2: Phylogenetic trees showing the coevolution of (A) ArsA and (B) ArsB homologues, (C) ArsA and (D) Acr3 homologues, and (E) ArsA and (F) ArsP homologues, encoded within the same genomes. For these trees, we use the following system of nomenclature to better designate the added protein pairs not found within the same operons. The first digit indicates separate organisms with the same three letter code (ex: Aaa1, Aaa2), the second digit indicates paralogues, if present (ex: Aaa1-1, Aaa1-2). If the transporter is located within the same genome, but not the same operon as the *arsA*, the letter E, standing for elsewhere, follows the name (ex: Aaa1-3E).

(A) ArsA (B) ArsD

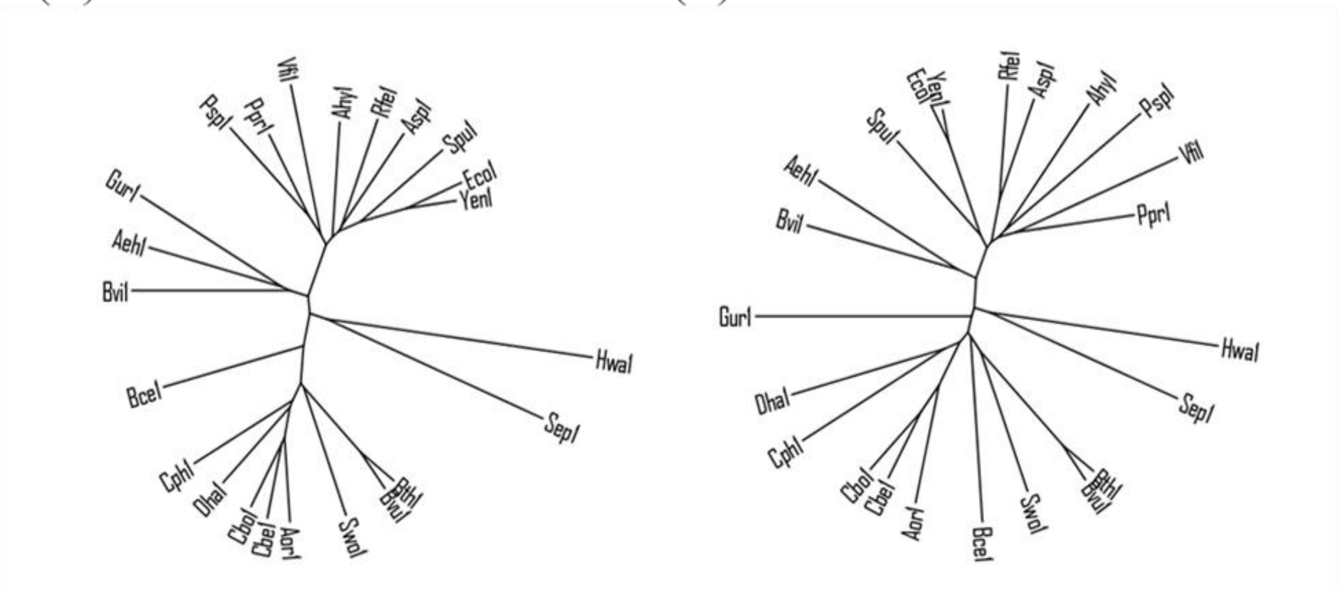

Figure S3: Phylogenetic trees showing the coevolution of (A) ArsA and (B) ArsD homologues encoded within the same operon. The methodology was as described in Fig. 1.
